# Investigation of the Influence of Anti-Wear Coatings on the Surface Quality and Dimensional Accuracy during Finish Turning of the Inconel 718 Alloy

**DOI:** 10.3390/ma16020715

**Published:** 2023-01-11

**Authors:** Krzysztof Smak, Piotr Szablewski, Stanisław Legutko, Bartłomiej Krawczyk, Edward Miko

**Affiliations:** 1 Faculty of Mechanical Engineering, Poznan University of Technology, 3 Piotrowo Street, 60-965 Poznan, Poland; 2 Pratt & Whitney Kalisz, 4a Elektryczna Street, 62-800 Kalisz, Poland; 3 Institute of Gears Research Excellence Center, The President Stanislaw Wojciechowski Calisia University, 4 Nowy Świat Street, 62-800 Kalisz, Poland; 4 Faculty of Mechatronics and Mechanical Engineering, Kielce University of Technology, Tysiąclecia Państwa Polskiego 7, 25-314 Kielce, Poland

**Keywords:** Inconel 718, turning, surface roughness, tool wear, microstructure

## Abstract

This piece of work deals with the influence assessment of the kind of coating of the cutting inserts and their wear on the dimensional accuracy and the top layer microstructure and roughness of the surface machined with constant cutting parameters *v_c_* = 85 m/min, *f* = 0.14 mm/obr and *a_p_* = 0.2 mm. The tests were performed on shafts made of Inconel 718 material under the conditions of finish turning, requiring a tool life of more than 20 min. The cutting inserts of identical geometry made of fine-grained carbide covered with coatings were applied by the PVD and CVD method. The values of the obtained diameter dimensions were assessed in reference to the assumed ones, as well as the values of the surface roughness and stereometry and the microstructure of the top layer. The nature and mechanisms of edge wear and its value expressed by the *VB_C_* parameter were also assessed. It was determined in the tests that the machined surface quality defined by the *Ra* and *Sa* roughness parameters and the dimensional accuracy were influenced not only by the coating microhardness but also by the method of applying the given coating. The lowest values of the tested roughness parameters were observed for the surface machined with an edge, with the S205 coating applied by the CVD method, which was characterized by the lowest microhardness. The edge with this coating also showed the lowest wear, defined by the *VB_C_* parameter, which translated into dimensional accuracy. Furthermore, the edge with the S205 coating also provided the best results with regard to the surface layer microstructure. The least favorable results, both in terms of dimensional accuracy and surface roughness, were obtained for the surface machined with a 1115-PVD-coated edge. The highest wear value was also recorded for this edge.

## 1. Introduction

Inconel 718 is a high-strength material and it belongs to the group of heat resistant alloys based on nickel [1]. Due to its mechanical and physical properties, such as high strength and creep resistance at high temperatures, it is widely applied in the aircraft industry for manufacturing parts working in the hot section of turbine engines [2]. Since Inconel 718 also shows low thermal conductivity [3,4] and the ability to be strain hardened, it is considered to be a hard-to-machine material. Due to the cost, the material is only used where it is required by the working conditions; therefore, elements made of the Inconel 718 alloy require the provision of the stable process of machining [5].

The material is frequently investigated and, despite growing knowledge about it, manufacturing parts of that material still bring many problems.

When machining this alloy, it is often a challenge to ensure the required surface finishing and to obtain the required dimensional accuracy [6,7]. In the case of turning, adequate cutting parameters and dedicated tools influencing the roughness and accuracy of the obtained dimensions can be applied [8]. The assessment of the machined surface can be performed examining the parameters of its roughness and stereometry, as well as by measuring the obtained dimensions. However, it should be taken into consideration that, when machining the Inconel 718 material, high temperature is generated in the cutting zone, which, combined with the mechanical load of the blade, often results in quick wear of the cutting edge of the tool [9].

Companies producing parts for aircraft engines permanently optimize their machining processes, both in respect of quality and reduction in the manufacturing costs. Due to the decreasing availability of qualified staff, the machining processes are increasingly elaborated according to the philosophy of “closed door machining”, which means the execution of parts without the operator’s interference during the operation. The creation of the process of manufacturing such components made of the Inconel 718 material must include the necessity to control the tool life throughout its operation [10,11]. Failure to adhere to this criterion may result in a condition in which it will be impossible to obtain the required surface quality and the top layer features, as well as the dimensional and shape accuracy. Moreover, prematurely worn tools can bring residual stresses to the structure of the machined surface. Devillez et al. [12] have shown in their work that the AlTiN-coated tool, due to its high hardness (3300 HV 0.05) compared to other coated tools, was characterized by good anti-friction properties and lower abrasive wear. The authors of the work noted, however, that due to the short cutting time, only the first stage of wear was examined. At this stage, flank wear, crater wear and notch wear were observed. In contrast, Zhao et al. [13] showed that increasing the Al content in the TiAlN coating reduces friction during dry machining.

Costes et al. [14] studied the effect of cutting speed on wear symptoms of CBN cutting inserts. They showed that at higher cutting speeds, i.e., above *v_c_* = 250 m/min, dominant wear mechanisms during the turning process are adhesion, then diffusion and finally abrasion. D’Addona et al. [15] investigated the effect of high cutting speeds on tool wear and surface roughness when machining Inconel 718 alloy with CVD-coated carbide inserts. The range of tested speeds reached *v_c_* = 255 m/min. When cutting with the highest cutting speeds used in the research, the authors of the work observed a much higher temperature in the cutting zone. This temperature causes much greater wear on the cutting edge, which is 3.5 times greater than at speeds of *v_c_* = 90 m/min and *v_c_* = 105 m/min. At high cutting speeds of *v_c_* = 255 m/min and *v_c_* = 190 m/min, in addition to very high flank wear, burns were also recorded. However, at speeds of *v_c_* = 90 m/min and *v_c_* = 105 m/min, the observed wear is uniform along the radius of the cutting insert corner, and the peeling of the coating was also observed. It should be noted that in this work, the length of the cutting path was 28 m, while the authors of this paper conducted research in which the length of the cutting path was 1600 m.

Liu et al. [16] applied carbide indexable inserts with AlTiSiN coating in their study when machining Inconel 718 alloy with HPC (high pressure coolant). The coolant pressures used were 3, 7, 15 and 20 MPa. It was observed that a change in the coolant pressure, with constant cutting conditions, affects the wear value of the cutting edge.

All the previously mentioned papers on the wear of cutting edges when machining Inconel 718 alloy indicate that the properties of this alloy, such as work hardening and low thermal conductivity, are the reason for the rapid wear of the cutting edges. Due to the low thermal conductivity of Inconel 718, a large part of the generated heat is transferred to the cutting tool, which, according to researchers, is the main cause of tool wear, and the hardened surface after the previous machining is responsible for the appearance of notch wear. Generally typical wear mechanisms observed for carbide inserts was intensive BUE leading to the chipping of the cutting tool edges. Tool companies specializing in the production of tools for machining aircraft alloys assume insert durability of 15 min. Of course, the tool life depends on the configuration of the part under machining and on the stiffness of the MFPT (machine, fixture, part, tool) system. In the case of thin walled parts, vibration often occurs during machining and significantly deteriorates the tool durability.

Tool life investigations are performed both for inserts with anti-wear coatings and without them. With regard to the finish machining of alloys on nickel matrix, the most frequently used tools are coated ones of sintered carbides [17]. The coatings are usually applied by the method of CVD (chemical vapor deposition) or PVD (physical vapor deposition). A coating applied by the CVD method is thicker, as compared to those applied by the PVD method, and is undoubtedly more resistant to abrasion at high temperatures. Tools with this type of coating are used when aiming at better durability of the blade but under the conditions of stable machining. Coating applied by the PVD method is one of less thickness, but it has both high resistance to abrasion and impact strength depending on its modification [18].

The application of the adequate insert coating and selection of correct cutting parameters, such as cutting speed, feed and cutting depth, have crucial influence on the quality of the machined surface and the dimensional accuracy, which we can assess by measuring roughness and examining the top layer microstructure, as well as by checking the obtained dimensions [19,20]. The quality of the machined surface in a cutting process is most often assessed by means of the roughness parameters of *Ra* and *Rz* [21]. With regard to feed, we can assess its influence on the values of the roughness parameters [22] using the Formula (1):

(1) $$Rz=\frac{f^{2}}{8\cdot r_{\varepsilon }}$$

where *f*—feed and *r*_ε_—corner radius.

It is a simplified formula on the basis of which we can predict the value of the *Rz* parameter, and we can also estimate the value of the *Ra* parameter according to the relationship:

(2) Ra=0.2556·Rz

The work presents the results of turning objects with tools of identical geometry but with different anti-wear coatings. The influence of the tool coating on the surface roughness and top layer microstructure, as well as the obtained dimension accuracy, has been assessed.

## 2. Materials and Methods

**Figure 1:**
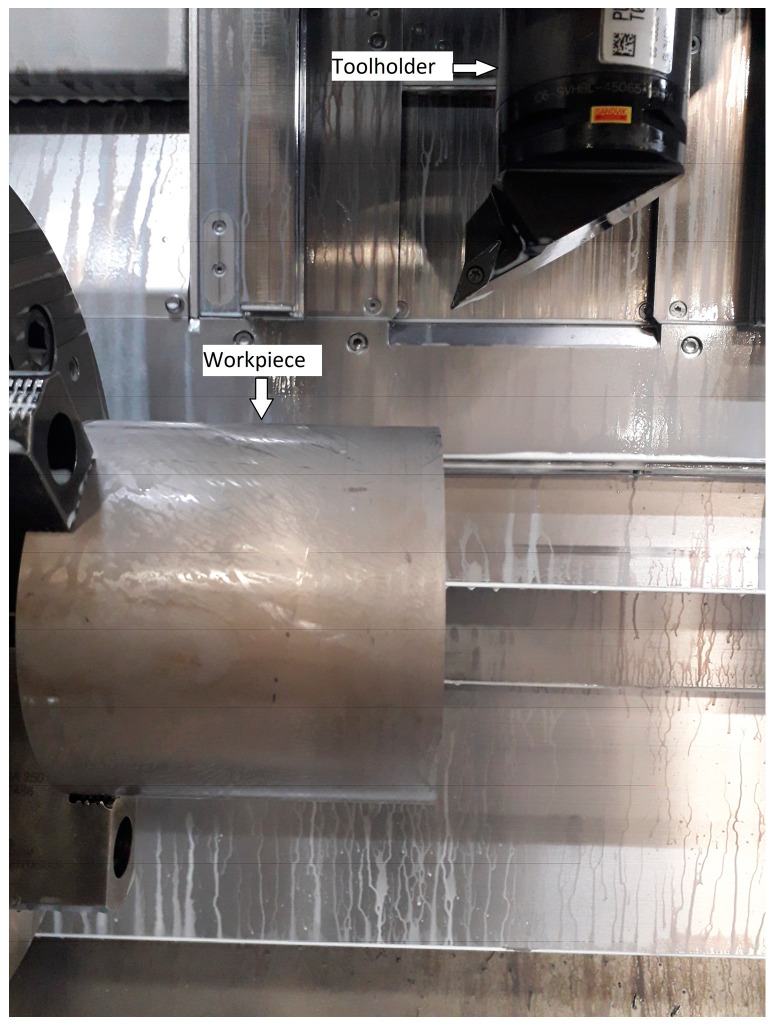
The tool and sample applied for the tests.

The tests were performed on a multi-task turning and milling centre made by WFL company. The material used in the investigation was Inconel 718 in aged condition with the hardness of 45 ± 2 HRC in the form of a shaft. The samples had an outer diameter of 132 mm and a length of 122 mm. Four positive cutting inserts made of sintered carbides by Sandvik with the geometry of VBGT 160408-UM with a corner radius of 0.8 mm were applied. The 1105 (TiAlN) and 1115 (TiAlN +TiAlN) coatings of the inserts were applied by the PVD method and their thickness was 2 µm. S05F (TiCrN + Al_2_O_3_ + TiN) and S205 (TiCN + Al_2_O_3_ + TiN) were coatings applied by the CVD method with a thickness of 4 µm. According to the information available in the producer’s catalogue [23], the 1105, S05F and S205 coatings have higher abrasion resistance and lower impact strength. The 1115 coating, on the other hand, has less abrasion resistance but higher impact strength. The authors of the work aimed to check the resistance of coatings applied by CVD and PVD methods to the tool life of cutting inserts and to the roughness of the machined surface. The VBGT 160408 insert with the UM rake face geometry was previously known to the authors of the work. Coatings 1105, 1115, S05F and S205 have different thicknesses, as evidenced by the rounding radius of the cutting edge *r_n_*. All cutting inserts were made to be the same size as grain cemented carbides, so we were able to assume that anti-wear coatings were responsible for the wear value on individual edges. The tests were performed in conditions allowing for the use of high pressure of the cooling lubricating liquid, Ecocool Global 10, produced by Fuchs oil Corporation. It is a concentrate of 8% emulsion based on mineral oil and 92% water. This kind of cooling and lubricating liquid is used in the aircraft industry. Holder-type Capto C6-SVHBL-45065-16HPA (Figure 1) was used. The cutting parameters were selected based on former tests: *v_c_* = 85 m/min; *f* = 0.14 mm/rev; *a_p_* = 0.2 mm. Formulas (1) and (2) were used to determine the value of the *Ra* roughness parameter, which was the factor determining the tests. The pressure of the cooling and lubricating liquid was 80 bar.

The tests were performed applying longitudinal turning of the outer diameter. This work was an introduction into investigations aimed at the elaboration of a technological solution for turning surfaces, which require long cutting paths, with tools of long reach (10 × D), and that is why 14 passes with a length of 40 mm were performed with each insert, reflecting the required path of one tool pass. For each tool, 4 measurements of the obtained diameters after purposefully determined passes were performed, which also reflects the check points for the machined surface, the cutting path of which has been projected. Technological requirements of the machined surface were *Ra =* 1.6 µm, while the hole diameter tolerance was ±0.05 mm.

**Figure 2:**
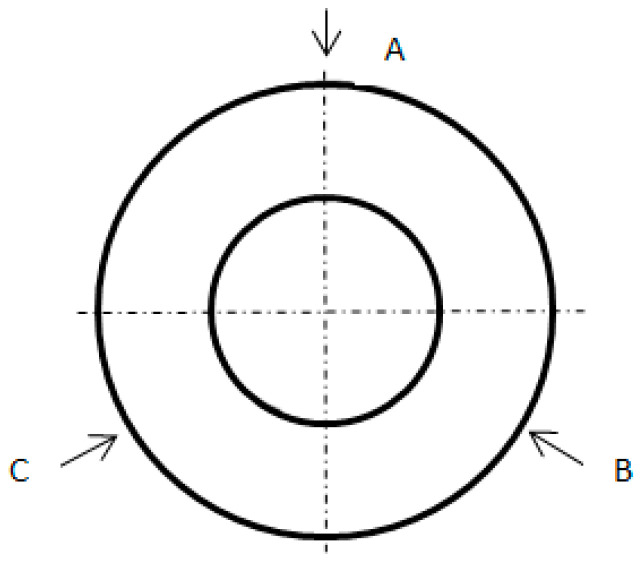
Locations of surface roughness and topography measurement.

**Figure 3:**
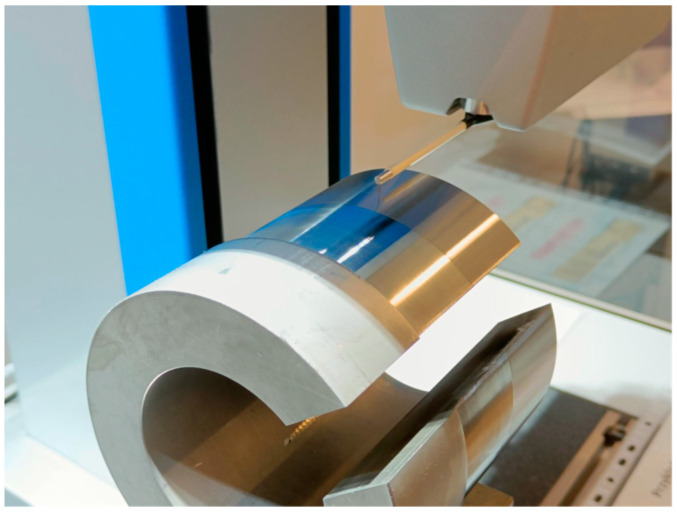
Measurement of the surface topography.

The surface roughness was assessed using the *Ra* parameter, which was measured for each sample at three places on the diameter; designated A, B and C; and distributed at 120 degrees intervals using a profilographometer, Surfcom 130A made by Seimitsu company (Figure 2). The roughness parameters were measured at l_r_ = 0.8 mm and l_n_ = 4.8 mm. According to the above schedule, the topography of the surface machined with the device Nanoscan 855, made by Jenoptik (Hommel-Etamic), was also measured and defined with the *Sa* parameter (Figure 3). The topography parameter was measured on an elementary surface, dimensioned as 5.21 mm and 4.95 mm, on which 200 passes at a measurement speed of 0.5 mm/s were performed.

The radius of the cutting edge rounding of the new insert and the wear of the working blade were measured using an optical microscope, 3D Portable RL of Bruker Alicona company. The measurements were performed with the objective magnification of 10×, where the transverse measurement area was 4 mm^2^ and the best transverse topographic resolution is 2 µm. The obtained diameter dimensions were measured in two cross sections by means of an electronic micrometer No.: 293-251, with a measurement range of 125–150 mm of Mitutoyo company. Photographs of the microstructures were obtained by means of an EPIPHOT 200 microscope from the Nikon company, with a magnification of 500×. The samples were melted in duracryl and etched with the Kalling’s reagent after polishing.

On the inserts use for the tests, microhardness of the coatings was measured. This was effected by means of a nanohardness meter, Picodentor Fischer HM500, whose loading range was 0.005–500 mN, and the range of tested hardness was 0.001–120,000 N/mm^2^.

The average values of the coating microhardness were:

- 1115—2574.14 HV;
- 1105—1978.81 HV;
- S05F—1777.09 HV;
- S205—1614.83 HV.

## 3. Results and Discussion

### 3.1. Influence of the Tool Coating on Dimensional Accuracy

The dimensional accuracy and the quality of the machined surface have been assessed depending on the coating applied on the cutting edge and on the wear of the cutting insert itself.

The surface quality has been assessed with the use of the roughness parameter, *Ra*, and the topography parameter, *Sa*. The accuracy of the obtained measurement has been checked by measurement of the diameter by means of an electronic micrometer. Since each cutting insert has performed 14 passes, a cutting path of 1620 m has been obtained [24]. For the adopted machining conditions, the working time of each tool was 19 min. The measurement of the diameter was purposefully performed after passes 2, 5, 9 and 14 (Table 1). Analyzing the influence of the anti-wear coating on the dimensional accuracy during longitudinal turning, one can see that in the range of higher abrasion resistances, the S205 coating applied by the CVD method ensures the best accuracy of the obtained dimensions.

**Figure 4:**
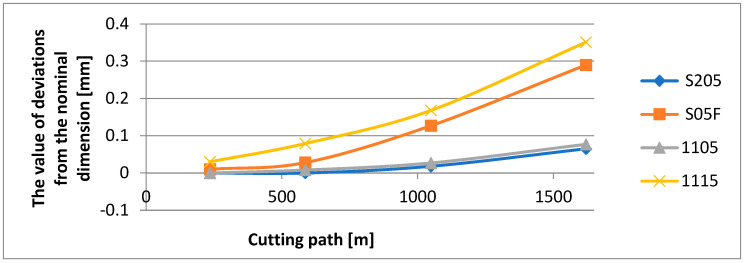
The value of deviations from the nominal dimension depending on the cutting path.

It should be pointed out, however, that this is the newest coating elaborated by the Sandvik company that is dedicated, first of all, to machining heat-resistant alloys. The lowest accuracy of the dimensions have been obtained for the 1115 coating in the range of the lower abrasion resistance applied by the PVD method (Figure 4).

### 3.2. Influence of the Tool Coating on the Surface Roughness and Surface Topography

**Figure 5:**
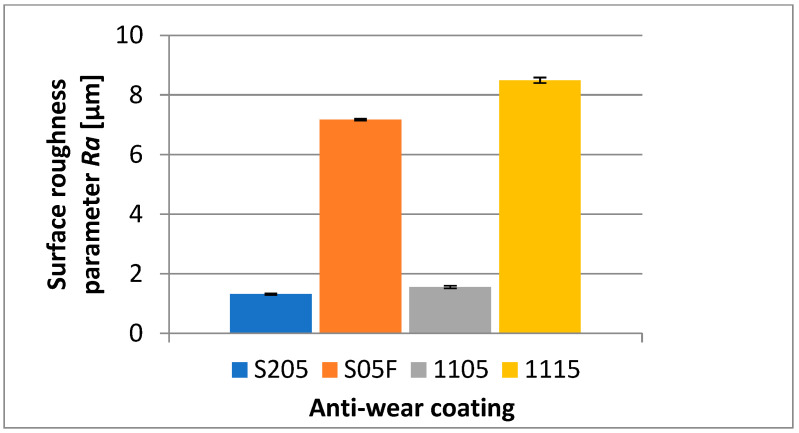
The influence of the cutting insert coating on the average value of the surface roughness parameter, *Ra*, of the machined surface.

Analyzing the influence of the cutting insert coating on the roughness parameter, *Ra*, one can see that the lowest values of that parameter have been recorded for the surface machined with the cutting insert with CVD S205 coating, for which the average value of *Ra* = 1.317 µm. A positive result is also obtained in the surface machined with the cutting insert with hard coating, PVD 1105, whose average roughness value has been obtained at a similar level of *Ra* = 1.553 µm. The high value of the parameter, *Ra* = 7.17 µm, for the surface machined with S05F coated cutting insert is curious because this is a coating with relatively high abrasion resistance (Figure 5).

**Figure 6:**
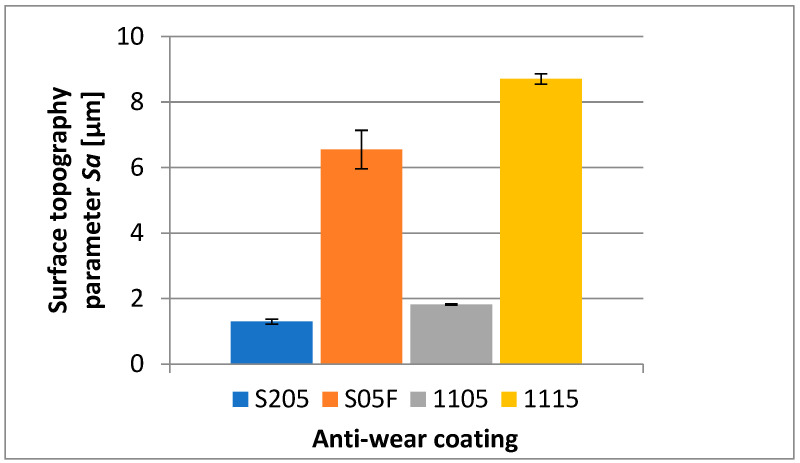
The influence of the cutting insert coating on the average value of the *Sa* topography parameter of the machined surface.

Considering the influence of the cutting insert coating on the surface topography assessed by the *Sa* parameter, one can see that the measurement results are similar to those of the surface roughness [25]. The lowest value of this parameter has also been obtained for the surface machined with an S205-coated cutting insert, and its average value is *Sa* = 1.293 µm. The highest average value of that parameter has been obtained for a surface machined with 1115-coated cutting insert, and it is *Sa* = 8.7 µm (Figure 6).

**Figure 7:**
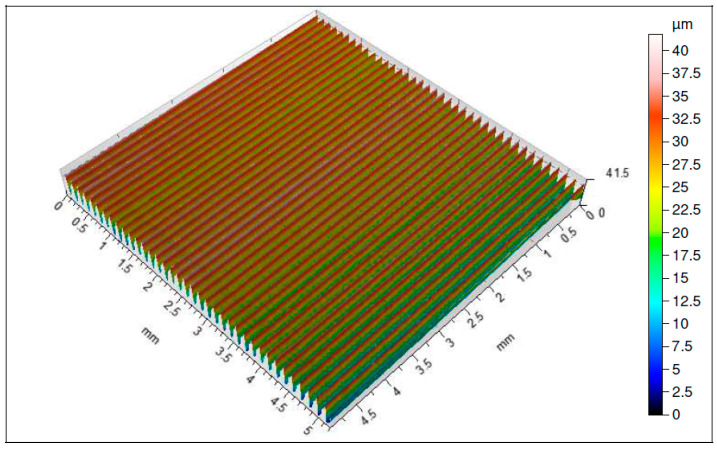
Topography of a surface obtained with an 1115-coated cutting insert.

**Figure 8:**
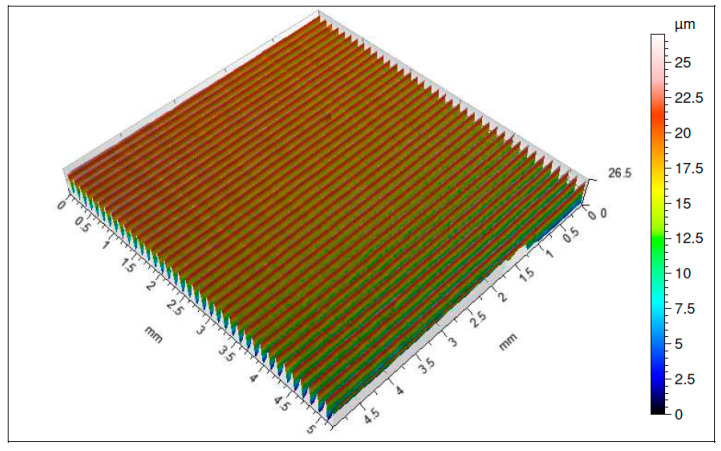
Topography of a surface obtained with an S05F-coated cutting insert.

**Figure 9:**
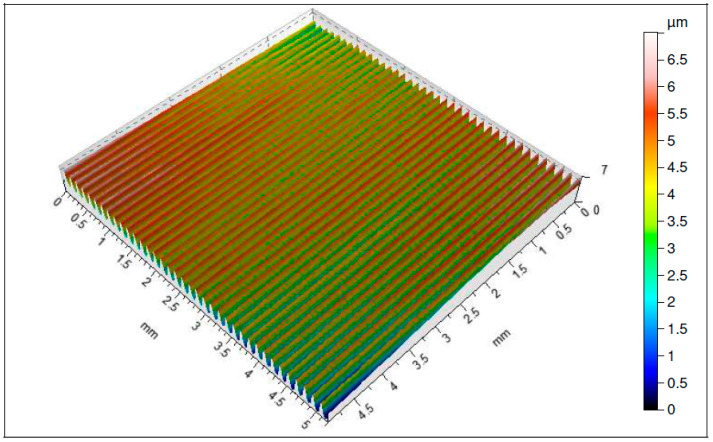
Topography of a surface obtained with an S205-coated cutting insert.

**Figure 10:**
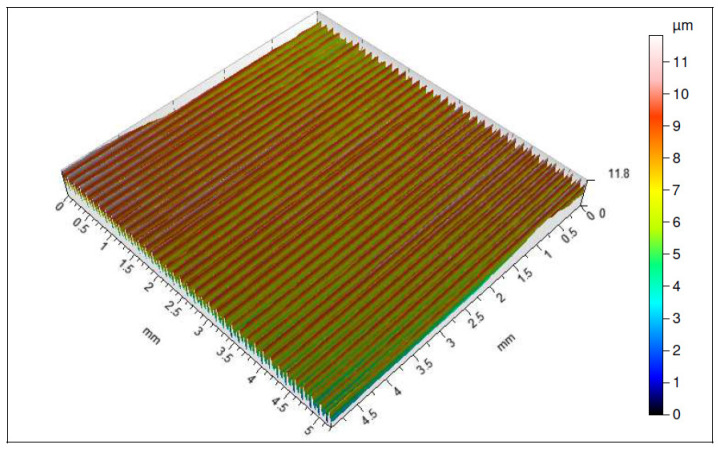
Topography of a surface obtained with an 1105-coated cutting insert.

It should be pointed out that both the coatings applied by the CVD and by PVD methods of the range of higher abrasion resistances significantly prevent intensification of the cutting insert wear, which has great influence on the quality of the machined surface (Figure 7, Figure 8, Figure 9 and Figure 10). All the analyzed isometric maps of the examined surfaces are characterized by an even distribution of valleys and ridges. These surfaces have different heights of micro-roughness. The highest value of the maximum height of the surface *Sz* = 41.8 µm was recorded for the surface machined with the 1115-coated edge (Figure 7). The parameters *Sp* and *Sv*, i.e., the maximum height of the surface peaks and the maximum height of the surface depressions, have equal values and amount to 20 µm. Another analyzed surface was obtained after machining with the S05F-coated insert (Figure 8). For this surface, the parameter *Sz* = 26.6 µm and the parameters *Sp* = 14.1 µm and *Sv* = 12.5 µm. The edges with the 1115 and S05F coatings generated surfaces for which the highest values of roughness parameters have been observed, and they have also been characterized by the highest *VBc* wear values (the subject of wear of the cutting blades is discussed later in the work). Cutting inserts with coatings S205 and 1105, on which approximately 50% less wear has been registered compared to edges with coatings 1115 and S05F, generated surfaces whose roughness parameters are several times lower. The smallest values of the parameters *Sz* = 7.01 µm, *Sp* = 3.84 µm and *Sv* = 3.17 µm have been recorded for the surface obtained with the S205 edge (Figure 9), for which the wear value of the insert was the lowest. Slightly higher values of the parameters *Sz* = 11.8 µm, *Sp* = 6.01 µm and *Sv* = 5.81 µm have been observed for the surface machined with the 1105-coated insert (Figure 10), for which the recorded wear of the *VBc* edge was 40 µm higher compared to the S205 coated edge. For the three tested surfaces, higher values of the *Sp* parameters than the *Sv* parameters have been observed, and, therefore, these surfaces are characterized by higher values of the maximum heights of the surface peaks. These are surfaces obtained after machining with S205-, 1105- and S05F-coated edges. Comparing the values of roughness parameters recorded on the surfaces after machining with edges with coatings applied with the same tool-coating method, i.e., CVD (S05F, S205) and PVD (1105, 1115), it can be seen that these parameters obtained after machining with an edge with S05F coating are more than 3.5 times greater than when machined with an S205-coated edge. The situation is similar for the 1115 and 1105 coatings. The roughness parameters recorded for the surface obtained with the 1115-coated edge are also 3.5 times higher than for the 1105 coating.

### 3.3. Influence of the Tool Coating on Its Wear

**Figure 11:**
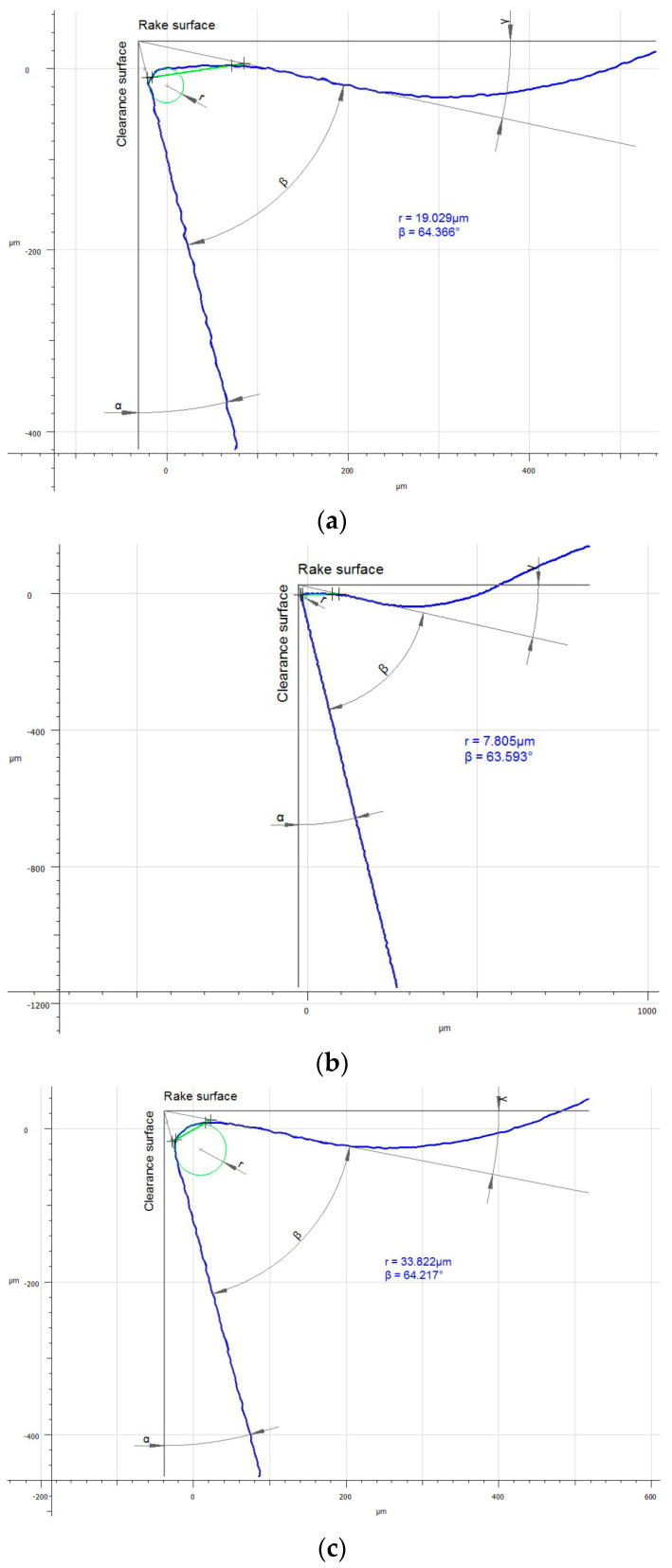

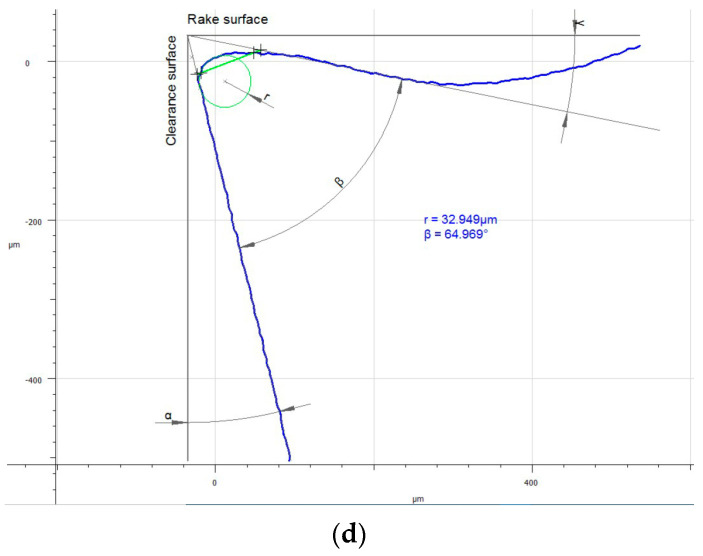
Comparison of the micro geometry of the cutting edges based on the radius of the cutting edge rounding, *r_n_*: (**a**) cutting insert with 1105 coating; (**b**) cutting insert with 1115 coating; (**c**) cutting insert with S05F coating; (**d**) cutting insert with S205 coating.

**Figure 12:**
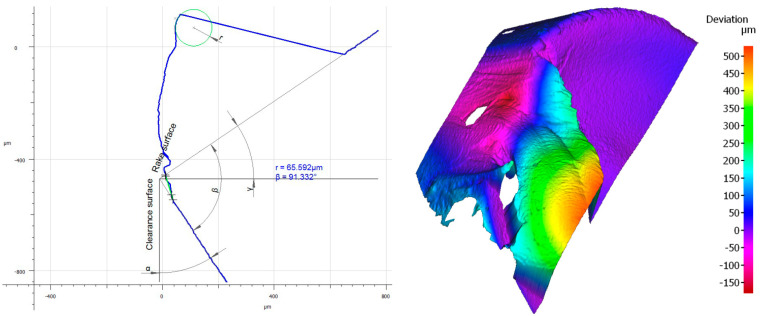
Topography and cross section profile of the cutting edge of a worn cutting insert with the S05F coating after 19 min of machining.

**Figure 13:**
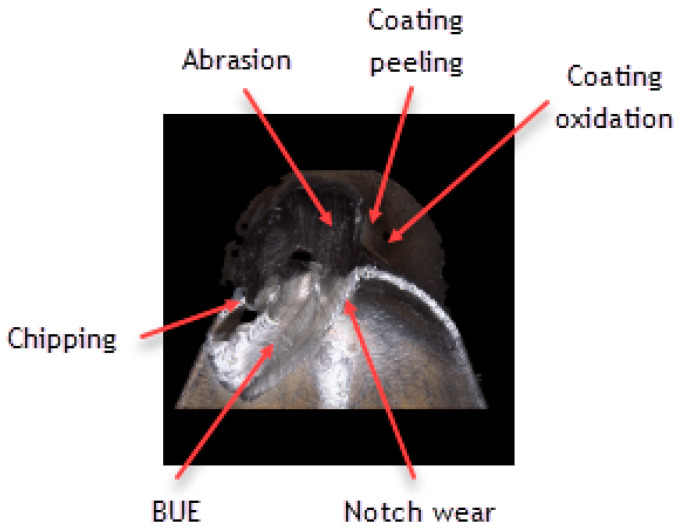
View of the cutting edge of a worn cutting insert with the S05F coating.

Analyzing the influence of the cutting insert coating on its wear, one should keep in mind that each insert has gone a very long cutting path, considering the material in which it had been working. It should also be noticed that the method of applying the given coating influences its thickness and, consequently, the cutting insert micro geometry. This can be seen, particularly, when comparing the value of the cutting edge rounding radius of the cutting inserts used in the investigation (Figure 11). Considering the width of the wear land on the flank face assessed by the *VB_c_* parameter, as well as appearance of the cutting insert itself, one can see significant difference between the coatings of the higher and the lower ranges of the abrasion resistance regardless of the method of applying the given coating. Undoubtedly, much wear has taken place on the cutting insert with the S05F coating where *VB_c_ =* 520 µm, which is astonishing because the coating is abrasion-resistant and is thicker due to the fact it is applied by the CVD method. The reason of such deterioration of the cutting insert could be of high value for the cutting speed adopted in the investigation and, since this is a slightly older kind of coating, consequently, in combination with the required cutting path, the abrasion and strength wear of the cutting insert has taken place (Figure 12 and Figure 13). There was also a significant loss of the cutting edge, on which micro-cracks and chipping have been noticed. The phenomenon of peeling and oxidation of the coating, particularly visible on the flank face, have also been observed.

**Figure 14:**
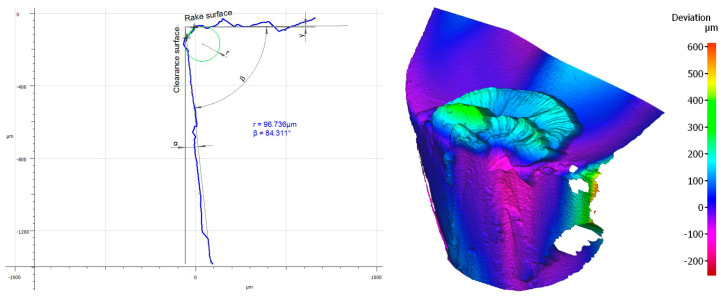
Topography and cross section profile of the cutting edge of a worn cutting insert with the 1115 coating after 19 min of machining.

**Figure 15:**
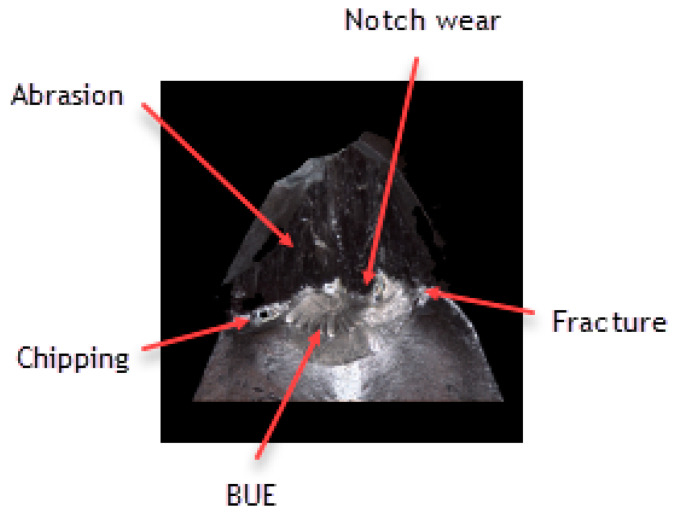
View of the cutting edge of a worn cutting insert with the 1115 coating.

It should also be noticed that a build-up has appeared on the cutting insert with S05F coating [26,27], which has probably been an effect of the Inconel 718 material susceptibility to the formation of it, as well as the increasing intensity of adhesion wear [28]. Analyzing the wear of the 1115-coated cutting insert for which *VB_C_ =* 540 µm, we can see that, in the operation of that tool, the wear mechanisms and kinds similar to those in the case of the cutting insert with the S05F have taken place (Figure 14 and Figure 15).

In the case of the 1115-coated cutting insert, one can say that we have to do with deterioration of the cutting edge. Drastic abrasion and strength wear in the form of chipping, notch and fracture have been noticed here. Nevertheless, it should be pointed out that it is a PVD coating, which has less thickness and lower abrasion resistance, as compared to the S05F. It should also be pointed out that the radius of the cutting edge rounding for the cutting insert with the 1115 coating was the lowest and amounted *r_n_* = 7.8 µm; this implies that the cutting insert was very sharp but had the lowest strength. Such a large wear was also due to the high cutting speed [29], as well as the length of the path that the tool had to pass.

**Figure 16:**
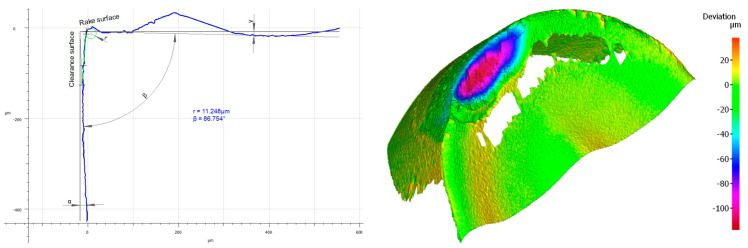
Topography and cross section profile of the cutting edge of the worn cutting insert with S205 coating after 19 min of machining.

**Figure 17:**
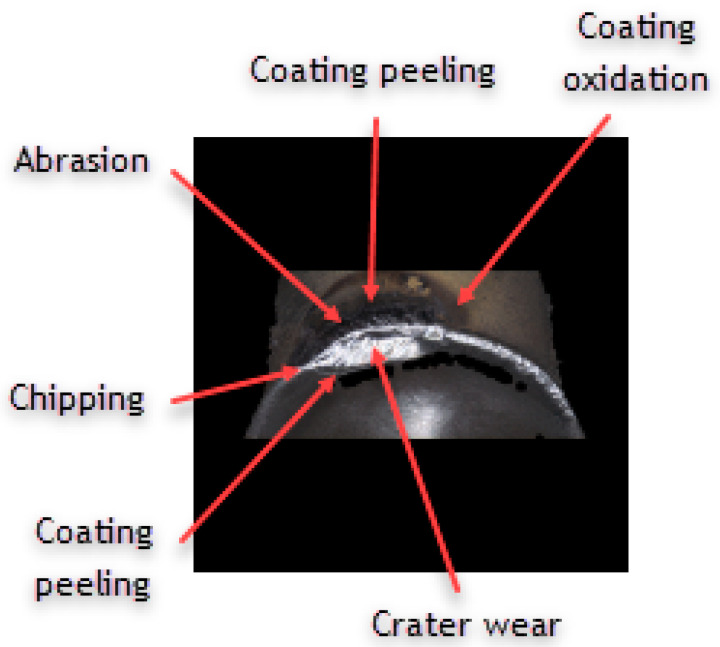
A view of the cutting edge of a worn cutting insert with S205 coating.

Assessing the wear of the cutting insert with S205 coating with *VB_C_* = 240 µm, one should emphasize that it is a very good CVD coating, having high abrasion resistance and resistance to high temperatures in the cutting zone [30] at relatively high cutting speeds [31,32]. The cutting insert wear resulting from the use of this tool presents an abrasive character. The crater wear, which has formed as result of the chip friction on the rake face, is characteristic of the Inconel 718 material (Figure 16 and Figure 17).

Wear mechanisms, such as chipping, peeling and oxidation of the coating, especially on the flank face, have also been observed. The S205 coating has endured the cutting conditions well considering the cutting insert wear, which has resulted in the accuracy of the obtained dimensions and the quality of the machined surface assessed by the *Ra* and *Sa* parameters.

**Figure 18:**
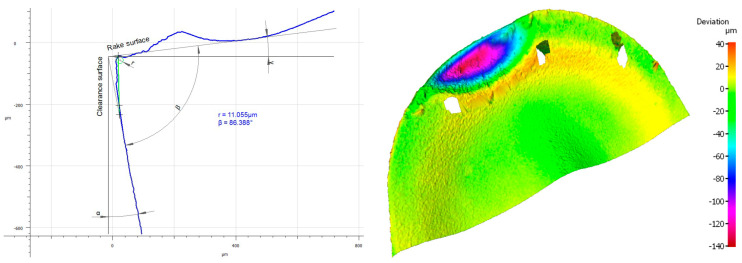
Topography and cross section profile of the cutting edge of the worn cutting insert with 1105 coating after 19 min of machining.

**Figure 19:**
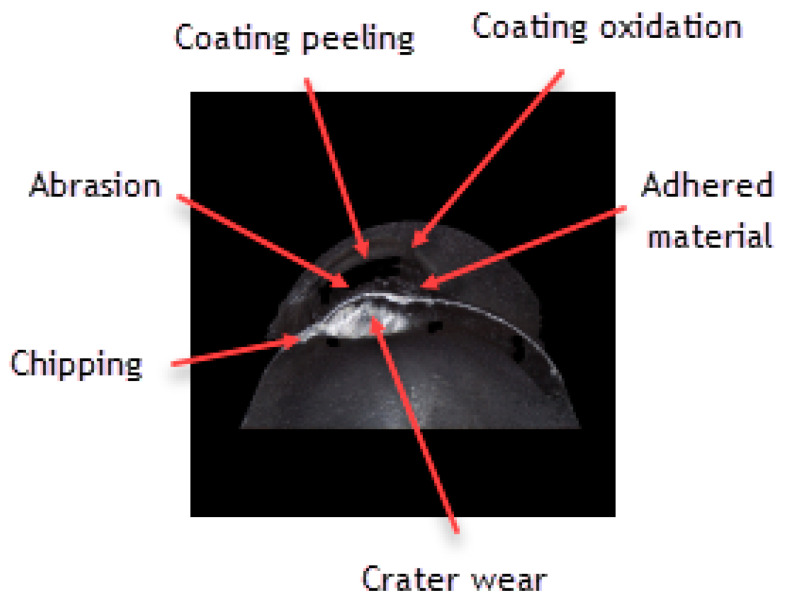
A view of the cutting edge of a worn cutting insert with 1105 coating.

The cutting insert with the 1105 coating with *VB_C_* = 280 µm also reveals low wear. Although the coating is applied by the PVD method, it has high abrasion resistance (Figure 18 and Figure 19). The cutting insert wear character is close to that which has appeared on the cutting insert with the S205 coating, in the form of a crater wear near the cutting edge stretching to the rake face. In the case of this edge, the abrasive nature of the wear has been noted. Nevertheless, chipping on the cutting edge, as well as peeling and oxidation of the coating, have also been recorded. In addition, material adhered to the flank face has been observed. The coating also ensures relatively high dimensional accuracy and surface quality, considering the cutting path it passes.

### 3.4. Analysis of the Microstructure of the Top Layer of the Machined Surface

The heat generated in the process of machining usually changes the alloy microstructure in the top layer, resulting in its deformations. Those deformations, in combination with high temperature, cause stresses, which can result in cracks.

The analysis of the microstructure has shown that the heat generated in machining has penetrated into the material under the process, resulting in changes to the microstructure in its top layer. In no cases has strain hardening been detected.

**Figure 20:**
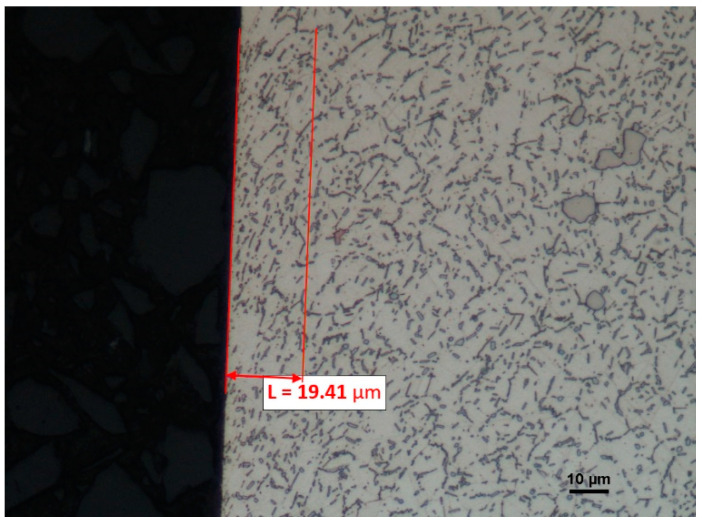
A view of the microstructure after longitudinal turning of the surface machined by the cutting insert with the S205 coating after etching.

Undoubtedly, the fewest changes to the top layer microstructure have taken place in the surface machined by the cutting insert with the S205 coating; they reach the depth of 19.41 µm but they are of mild character (Figure 20).

**Figure 21:**
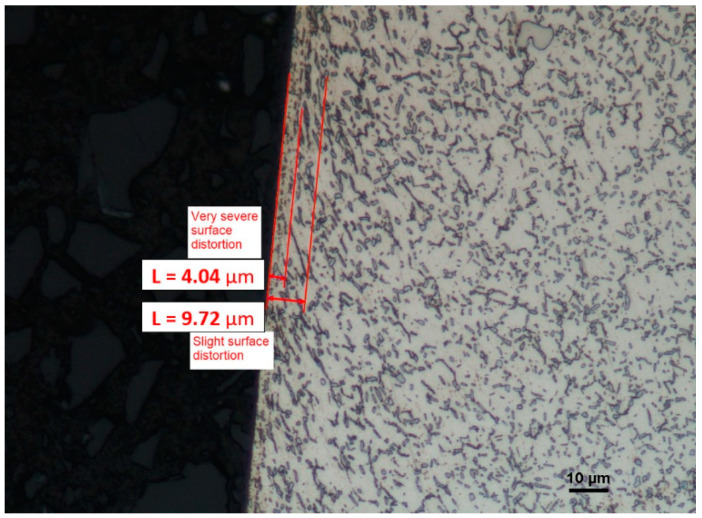
A view of the microstructure after longitudinal turning of the surface machined by the cutting insert with the 1105 coating after etching.

The microstructure of the top layer of a surface machined by the cutting insert with the 1105 coating shows greater changes, which, down to the depth of 4.04 µm, have the character of serious deformations. On the other hand, below that value, down to the depth of 9.7 µm, their character is mild (Figure 21). This can be due to the fact that this coating has less strength, as compared to S205, which causes higher temperature and stresses, resulting in the intensity of the deformations that arise.

Considering the changes to the microstructure of the surfaces machines by cutting inserts with the S05F and 1115 coatings, it should be pointed out that the deformations, which had taken place here, undoubtedly result from exceeding the permissible criterion of the blunting of both cutting inserts.

**Figure 22:**
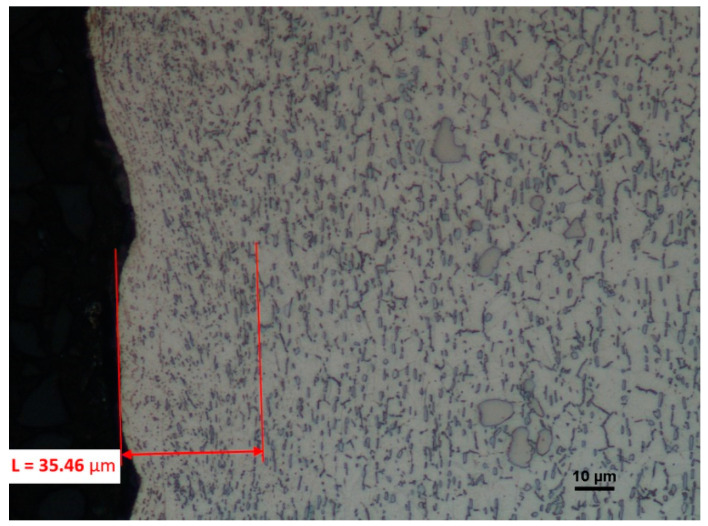
A view of the microstructure after longitudinal turning of a surface machined by a cutting insert with the S05F coating after etching.

In the case of the microstructure of the surface machined by the cutting insert with the S05F coating, the deformations are of serious character and reach depths of 35.46 µm (Figure 22).

**Figure 23:**
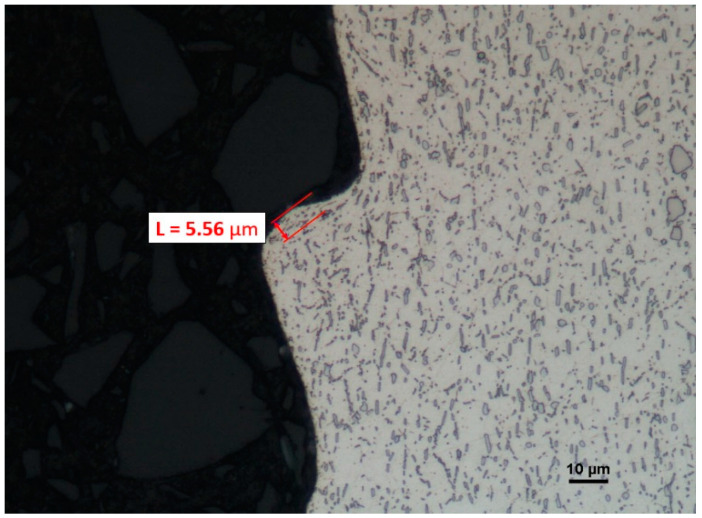
A view of the microstructure after longitudinal turning of a surface machined by a cutting insert with the 1115 coating after etching.

The changes to the microstructure of a surface machined by the cutting insert with 1115 coating reach a depth of 5.56 µm and also show serious deformation of grains (Figure 23).

## 4. Conclusions

Based on the investigation performed, the following conclusions have been formulated:

1. It has been observed that the microhardness of the anti-wear coatings applied on the cutting inserts is not the only factor determining the abrasion resistance in machining the Inconel 718 material.
2. It has been found that not only the abrasion resistance of the anti-wear coatings but also the method of their application influence the accuracy of the obtained dimensions and the surface quality assessed by the *Ra* and *Sa* parameters. The least deviation from the nominal dimension has been obtained for the cutting insert with the S205 coating applied by the CVD method. The deviation was 0.065 mm. The largest deviation from the nominal dimension has been obtained for the cutting insert with 1115 coating applied by the PVD method. This deviation was 0.351 mm. The lowest average values of the roughness parameters, *Ra* and *Sa*, have been obtained for a surface machined by a cutting insert with the S205 coating; the parameters were *Ra* = 1.317 µm and *Sa* = 1.293 µm. The highest values of those parameters have been obtained for the surface machined by a cutting insert with the 1115 coating; those values were *Ra* = 8.49 µm and *Sa* = 8.7 µm.
3. In machining the Inconel 718 alloy, the kind of the cutting insert coating applied influences the character and magnitude of the cutting insert wear, particularly when a long cutting path is required. The measurements of wear have shown that the strongest cutting insert is one with the S205 coating, however, the 1105 coating also shows very good strength properties. High wear has been recorded for the cutting insert with the S05F coating, which also has high abrasion resistance; nevertheless, the value of the cutting speed adopted in the investigation could be too high for this coating. The highest wear has been recorded for the cutting insert with the 1115 coating, which is justified because that coating shows less abrasion resistance. High cutting speed and long cutting path, in combination with a very sharp cutting edge, have contributed to the quick cutting insert wear.
4. The analysis of the microstructure of the top layer of the machined surface shows that the lowest depth of the deformation of grains, having a mild character, has also been obtained for a surface machined by a cutting insert with the S205 coating. The deepest deformation of grains, with serious character, has taken place on a surface machined by a cutting insert with the S05F coating.
5. It should be pointed out that the value of surface roughness does not always reflect the deformation of grains in the microstructure of the top layer. A machined surface for which the highest values of roughness parameters have been recorded did not have the largest grain deformation.

## Figures and Tables

**Table 1:** The values of diameter dimensions obtained in the investigation.

No. of the Tool Pass/Cutting Path [m]	Nominal Dimension [mm]	Average Values of the Dimension Obtained in Machining with the Cutting Insert with the S205 Coating [mm]	Average Values of the Dimension Obtained in Machining with the Cutting Insert with the S05F Coating [mm]	Average Values of the Dimension Obtained in Machining with the Cutting Insert with the 1105 Coating [mm]	Average Values of the Dimension Obtained in Machining with the Cutting Insert with the 1115 Coating [mm]
2/235	131.2	131.2 ± 0.002	131.21 ± 0.003	131.2 ± 0.002	131.23 ± 0.003
5/586	130	130 ± 0.002	130.028 ± 0.003	130.008 ± 0.002	130.079 ± 0.003
9/1049	128.4	128.418 ± 0.003	128.527 ± 0.004	128.427 ± 0.003	128.568 ± 0.004
14/1620	126.4	126.465 ± 0.003	126.69 ± 0.005	126.477 ± 0.003	126.751 ± 0.005

## Data Availability

Data sharing is not applicable to this article.
